# Foraging Signals Promote Swarming in Starving Pseudomonas aeruginosa

**DOI:** 10.1128/mBio.02033-21

**Published:** 2021-10-05

**Authors:** Divakar Badal, Abhijith Vimal Jayarani, Mohammad Ameen Kollaran, Deep Prakash, Monisha P, Varsha Singh

**Affiliations:** a Biosystems Science & Engineering, Indian Institute of Science, Bangalore, India; b Department of Molecular Reproduction, Development & Genetics, Indian Institute of Science, Bangalore, India; Emory University School of Medicine

**Keywords:** *Pseudomonas aeruginosa*, swarming motility, ethanol oxidation, starvation, foraging signal, rhamnolipid, *Cryptococcus neoformans*

## Abstract

The opportunistic human pathogen Pseudomonas aeruginosa is known for exhibiting diverse forms of collective behaviors, like swarming motility and biofilm formation. Swarming in P. aeruginosa is a collective movement of the bacterial population over a semisolid surface, but specific swarming signals are not clear. We hypothesize that specific environmental signals induce swarming in P. aeruginosa. We show that under nutrient-limiting conditions, a low concentration of ethanol provides a strong ecological motivation for swarming in P. aeruginosa strain PA14. Ethanol serves as a signal and not a source of carbon under these conditions. Moreover, ethanol-driven swarming relies on the ability of the bacteria to metabolize ethanol to acetaldehyde using a periplasmic quinoprotein alcohol dehydrogenase, ExaA. We found that ErdR, an orphan response regulator linked to ethanol oxidation, is necessary for the transcriptional regulation of a cluster of 17 genes, including *exaA*, during swarm lag. Further, we show that P. aeruginosa displays characteristic foraging motility on a lawn of Cryptococcus neoformans, a yeast species, in a manner dependent on the ethanol dehydrogenase ErdR and on rhamnolipids. Finally, we show that ethanol, as a volatile, could induce swarming in P. aeruginosa at a distance, suggesting long-range spatial effects of ethanol as a signaling molecule.

## INTRODUCTION

Adopting different modes of motilities befitting the prevailing niche conditions is a well-documented phenomenon in many bacterial species (1, 2). On a semisolid nutritionally defined surface, the opportunistic human pathogen Pseudomonas aeruginosa exhibits a characteristic flagellum-driven locomotory social trait called swarming (3). Swarming in P. aeruginosa is a quorum-dependent motility relying on Rhl and Las quorum-sensing (QS) systems (3). Over the past 2 decades, cellular and molecular regulatory aspects of P. aeruginosa swarming have been well examined. For instance, the requirement of self-synthesized biosurfactants, the rhamnolipids (4, 5), at the population level and c-di-GMP turnover at the cellular level have been well established (6, 7). However, specific environmental signals that serve as ecological motivations for swarming in bacteria remain a poorly understood facet of this form of collective behavior.

Previous studies indicated that iron or phosphate limitation could promote swarming in P. aeruginosa (8, 9). However, the requirement for as many as 44 two-component signaling system genes in swarming suggests the potential involvement of several extrinsic cues in promoting swarming in P. aeruginosa (10). Also, swarming in P. aeruginosa is typically observed on growth-limiting nutritionally deficient media, suggesting that the limitation of one or more nutrients might promote swarming. Do specific environmental signals induce the formation and extension of tendrils, a characteristic of P. aeruginosa swarming? It is not understood if the extension of tendrils is a form of chemotactic motility driving the bacterial population toward a nutrient, which is limiting. Previous observations in Escherichia coli swarming (11) suggest that swarming might be a chemotactic movement toward a source of limiting nutrients or a food source. If this is true, broad or nutrient-specific starvation might be essential for bacteria to engage in swarming.

Tendrils are a defining feature of the PA14 strain of P. aeruginosa on various media, although some strains, such as PAO1, can swarm without tendrils on fastidious anaerobe broth (4, 10, 12–16). Prior to tendril formation, swarm populations of P. aeruginosa PA14 typically exhibit 8 to 12 h of swarm lag (10). Though the swarm lag is usually much longer than the planktonic-phase growth lag, it is not clear whether the swarm lag is required just to build the cell density necessary for a quorum or whether it also prepares cells to achieve a swarm-proficient morphological or physiological state. Induction of starvation in P. aeruginosa can have a significant impact on its physiology, including its lifestyle. For instance, glucose depletion can induce biofilm dispersal in P. aeruginosa under regulation by cyclic AMP (cAMP) and c-di-GMP second-messenger molecules (17, 18). Similarly, phosphate starvation enhances swarming motility and virulence-associated traits in P. aeruginosa (8). However, the observed plasticity in P. aeruginosa swarming in different nutrient contexts (10) suggests that many signals, particularly specific nutrient limitations, might be responsible for the induction of swarming.

In this study, we investigated environmental cues that promote swarming motility in P. aeruginosa PA14 by comparing the transcriptome of the P. aeruginosa population during the early and late swarm lag. On a peptone-based swarm agar, transcripts for enzymes and several regulators involved in aerobic oxidation of ethanol were significantly upregulated under the control of the response regulator ErdR. Of these, the alcohol dehydrogenase ExaA, necessary for the conversion of ethanol to acetaldehyde, was essential for swarming. We found that exogenous ethanol served as a critical driver of swarming in P. aeruginosa under nutrient-limiting conditions. Ethanol-induced tendril formation in P. aeruginosa was also observed under other carbon-limiting-conditions mimicking starvation, suggesting that ethanol serves as a foraging cue for P. aeruginosa populations facing starvation. Using a lung-colonizing pathogenic yeast, Cryptococcus neoformans, we found that P. aeruginosa shows tendril-like chemotactic movement upon the yeast lawn in a manner that is partly dependent on ethanol oxidation and wholly dependent on rhamnolipids. In all, our study shows that ethanol acts as a foraging signal to induce swarming and foraging motility in the starving population of P. aeruginosa, allowing it to spread and find nutrition.

## RESULTS

### Aerobic ethanol oxidation is necessary for swarming in P. aeruginosa PA14.

**FIG 1:**
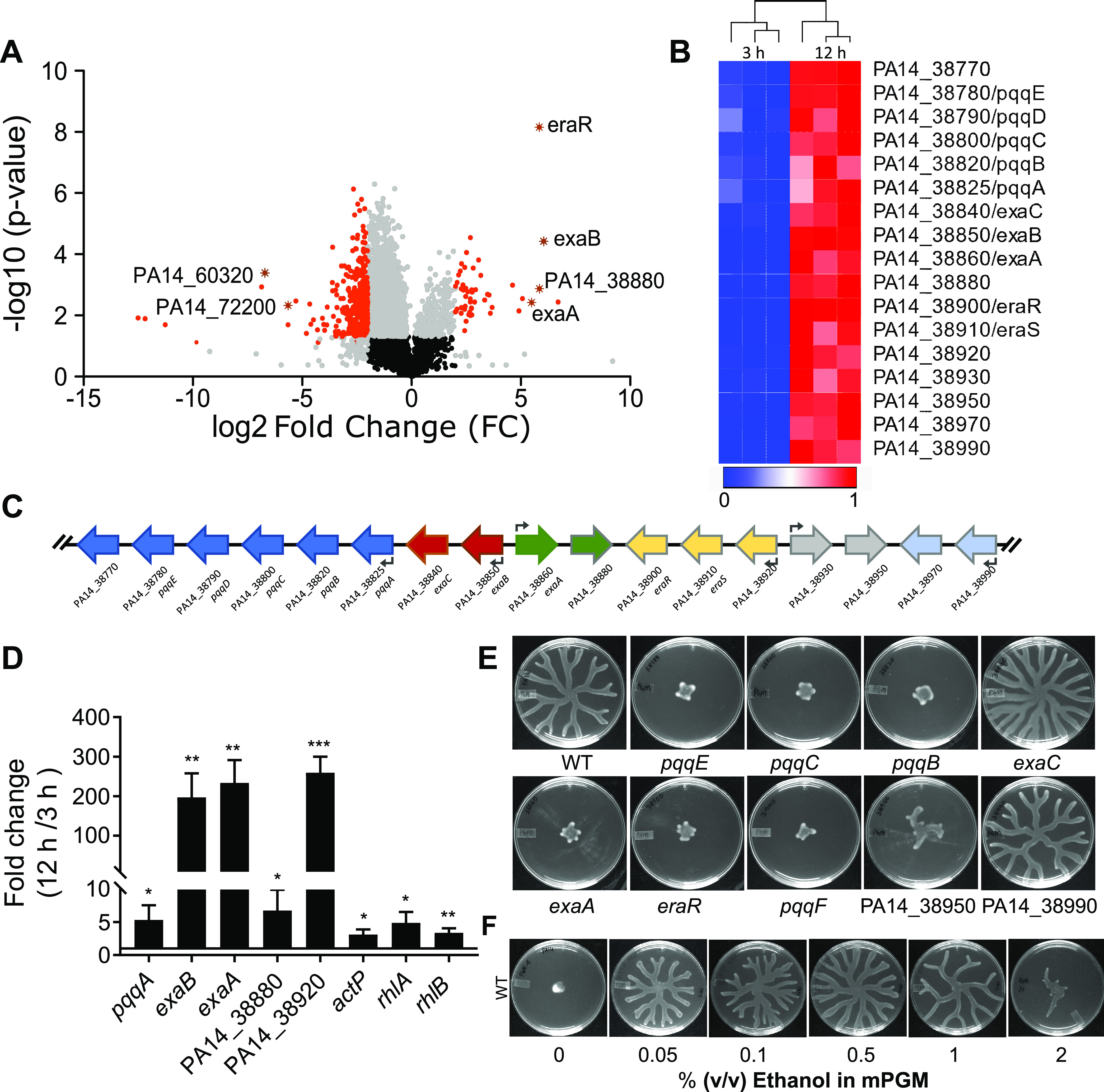
Ethanol oxidation is necessary for swarming in P. aeruginosa PA14. (A) Volcano plot highlighting genes dysregulated in the 12h swarm of P. aeruginosa PA14 over a 3-h swarm. Genes are indicated in black (nonsignificant; FC < 1.5, *P* > 0.05), gray (nonsignificant; FC > 1.5, *P* > 0.05), or red (significant; FC > 1.5, *P* < 0.05). Asterisks highlight a few randomly selected genes from the significantly dysregulated (red) group. (B) Heat map of 17 genes present in a cluster in PA14 genome in a 3h versus a 12h swarm. Columns represent independent RNA samples. (C) Organization of the 17-gene cluster in the PA14 genome. Colors represent various operons in the cluster. (D) qRT-PCR analysis of 5 genes from 17 gene clusters and *actP*, *rhlA* and *rhlB* transcripts in a 12h swarm over a 3h swarm. An unpaired *t* test was used for analysis of significance (*, *P* ≤ 0.05; **, *P* ≤ 0.01; ***, *P* ≤ 0.001). (E) Swarming phenotypes of strains with gene disruption in the 17-gene cluster on PGM (mPGM plus 0.1% ethanol) swarm agar. (F) Impact of increasing concentration of ethanol on swarming in WT P. aeruginosa on mPGM swarm agar.

To understand the extrinsic cues that induce swarming in P. aeruginosa populations, we sought to investigate the transcriptional response of bacteria during the swarm lag. We thus examined the transcriptome of P. aeruginosa PA14 on peptone growth medium (PGM) swarm medium, where it exhibits a long swarm lag of 12 h (10). Swarm lag is the time taken by the bacterial population to form tendrils after being spotted on a swarm surface. By using an RNA sequencing (RNA-seq) approach, we compared the transcriptome of a wild-type PA14 population early during the swarm lag (3 h after spotting) and late during the swarm lag (at 12 h after spotting). At 12 h in the swarm lag, the time point just before the induction of tendril formation, we found that 97 and 1,039 transcripts were significantly up- and downregulated, respectively, compared to the population at 3 h in the swarm lag (Fig. 1A; Table S1A). It was notable that a cluster of 17 genes was among the most upregulated transcripts at the end of the swarm lag (Fig. 1B and C). Some of the genes in the cluster have been previously characterized for growth when ethanol is the sole carbon source (19, 20). This cluster contains genes encoding a periplasmic quinoprotein, ethanol dehydrogenase (*exaA*), cytochrome *c*_550_ (*exaB*), acetaldehyde dehydrogenase (*exaC*), two response regulators and sensor kinases, and two hypothetical proteins and genes in the pyrroloquinoline quinone (PQQ) biosynthesis operon, *pqqABCDE*. PQQ is the prosthetic group required for the alcohol dehydrogenase activity of ExaA (20). A reverse transcription-quantitative PCR (RT-qPCR) analysis further confirmed that these genes were indeed upregulated at 12 h of swarm lag compared to 3 h (Fig. 1D).

10.1128/mBio.02033-21.8 TABLE S1 (A) Genes upregulated in P. aeruginosa PA14 on swarm agar at 12 h compared to PA14 on swarm agar at 3 h. (B) Genes upregulated in the P. aeruginosa *erdR* mutant on swarm agar at 12 h compared to PA14 on swarm agar at 12 h. (C) Strains used in the study. (D) Primers used in the study. (E) Plasmids used in the study. Download Table S1, XLSX file, 0.1 MB. Copyright © 2021 Badal et al. 2021 Badal et al. https://creativecommons.org/licenses/by/4.0/ This content is distributed under the terms of the Creative Commons Attribution 4.0 International license.

To test whether the oxidation of ethanol is necessary for inducing swarming, we examined the swarming phenotype of *exaA*, *pqqB*, *pqqC*, *pqqE*, *pqqD*, and *exaC* mutants retrieved from the Harvard Tn insertion mutant library for PA14 (21). We also checked the phenotypes of two response regulators, PA14_38950 and PA14_38990, in the cluster. Although *exaA* and its cofactor were found to be necessary for swarming, *exaC*, encoding the aldehyde dehydrogenase involved in the conversion of acetaldehyde to acetate, was completely dispensable for swarming (Fig. 1E). We discovered that *pqqF*, necessary for PQQ synthesis, was not present in the cluster, but it was also essential for swarming (Fig. 1E). This suggested that the conversion of ethanol to acetaldehyde is necessary for swarming, while the subsequent step, conversion of acetaldehyde to acetate, may be dispensable. Interestingly, an acetate permease gene (*actP*; PA14_22350) present outside the cluster was also found to be induced at 12 h of swarm lag. However, an *actP* mutant was efficient in swarming in response to ethanol (Fig. S1). In an additional experiment, we found that the replacement of ethanol by sodium acetate in PGM swarm agar could not induce swarming in PA14 (Fig. S1). These experiments suggested that acetaldehyde produced from ethanol, and not acetate, was required for inducing swarming in P. aeruginosa. Thus, ethanol is not a carbon source but a signal for swarming in P. aeruginosa.

10.1128/mBio.02033-21.1 FIG S1 Acetate permease mutant (*actP)* swarming on (A) mPGM agar and (B) mPGM agar supplemented with ethanol (0.1% [vol/vol]). Effect of acetate supplementation on swarming of the WT and the *erdR* mutant of P. aeruginosa PA14 shown (C) in a schematic, (D) on mPGM agar, and (E) on mPGM swarming agar with 0.1% sodium acetate. Download FIG S1, PDF file, 0.09 MB. Copyright © 2021 Badal et al. 2021 Badal et al. https://creativecommons.org/licenses/by/4.0/ This content is distributed under the terms of the Creative Commons Attribution 4.0 International license.

The peptone-based swarming medium PGM–0.6% agar contains 0.1% (vol/vol) ethanol as a solvent for cholesterol (10) (see Materials and Methods). We found that removal of ethanol completely abrogated swarming (Fig. S2), suggesting that exogenous ethanol can induce swarming in P. aeruginosa. Interestingly, the presence of trace amounts of ethanol has been reported to be present in cystic fibrosis lungs and to be produced by microbes, such as Cryptococcus neoformans, which interact with P. aeruginosa (22–24). A recent report indicated a negative impact of a high concentration (1%) of ethanol on swarming and swimming (25), while another study reported a positive impact of 1% ethanol on the biofilm formed by P. aeruginosa strain PAO1 (26). Thus, we examined the effects of various concentrations of ethanol on P. aeruginosa swarming and found that a concentration range of 0.05 to 0.5% (vol/vol) of ethanol induced swarming, while 1% and 2% ethanol caused reduced swarming (Fig. 1F), suggesting that ethanol has a dose-dependent effect on P. aeruginosa swarming. We used 0.1% ethanol as a swarm-promoting dose for the rest of this study, unless otherwise specified. We found that other low-molecular-weight alcohols, such as methanol and propan-2-ol, could not induce robust swarming in P. aeruginosa (Fig. S3A to D). However, the replacement of ethanol with acetaldehyde could induce swarming in P. aeruginosa (Fig. S3E), consistent with the requirement of the alcohol dehydrogenase gene *exaA* (Fig. 1E). Both *exaA* and *pqqB* were not required for acetaldehyde-induced swarming (Fig. S4A to C), as expected. Importantly, the acetaldehyde dehydrogenase ExaC was not necessary for acetaldehyde-induced swarming (Fig. S4D), suggesting that acetaldehyde may have a signaling function and that its conversion to acetate was not a prerequisite for swarming.

10.1128/mBio.02033-21.2 FIG S2 Swarming motility of P. aeruginosa on swarm agar (A), mPGM (B), and mPGM supplemented with cholesterol (dissolved in 0.1% ethanol) (C) with 0.1% ethanol. Download FIG S2, PDF file, 0.2 MB. Copyright © 2021 Badal et al. 2021 Badal et al. https://creativecommons.org/licenses/by/4.0/ This content is distributed under the terms of the Creative Commons Attribution 4.0 International license.

10.1128/mBio.02033-21.3 FIG S3 P. aeruginosa and *erdR* mutant swarming on (A) mPGM, mPGM supplemented with 0.1% ethanol, mPGM supplemented with 0.1% methanol, mPGM supplemented with 0.1% isopropanol, mPGM supplemented with 0.1% acetaldehyde. (B) WT and *erdR* strain swarming on mPGM supplemented with 0.1% of ethanol, isopropanol, methanol, sodium acetate, and acetaldehyde in *trans*, in tripartite plates. (G) WT and *erdR* strain swarming on mPGM supplemented with various concentrations of ethanol. Download FIG S3, PDF file, 0.2 MB. Copyright © 2021 Badal et al. 2021 Badal et al. https://creativecommons.org/licenses/by/4.0/ This content is distributed under the terms of the Creative Commons Attribution 4.0 International license.

10.1128/mBio.02033-21.4 FIG S4 P. aeruginosa swarming on mPGM supplemented with 0.1% acetaldehyde for (A) the WT, (B) the *exaA* mutant, (C) the *pqqB* mutant, and (D) the *exaC* mutant. Download FIG S4, PDF file, 0.05 MB. Copyright © 2021 Badal et al. 2021 Badal et al. https://creativecommons.org/licenses/by/4.0/ This content is distributed under the terms of the Creative Commons Attribution 4.0 International license.

Taken together, these experiments suggested that a low concentration of ethanol induces swarming in P. aeruginosa via its conversion to acetaldehyde by the specific alcohol dehydrogenase ExaA.

### The response regulator ErdR controls ethanol-induced swarming in P. aeruginosa.

**FIG 2:**
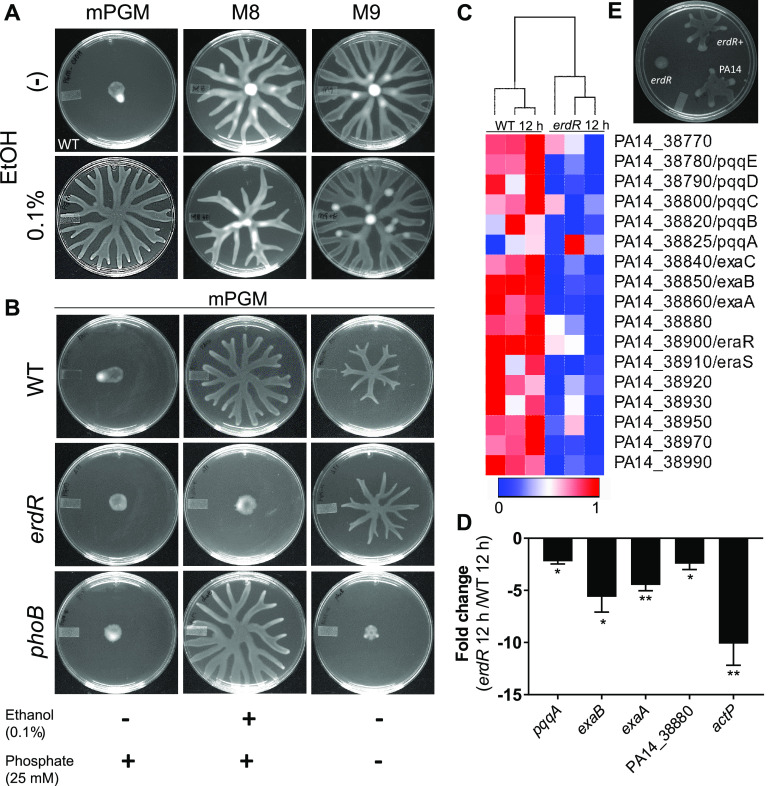
ErdR controls ethanol-induced swarming and transcriptional response during swarm lag. (A) Effect of low-concentration ethanol on PA14 (WT) swarming in mPGM, M8 and M9 swarm agar. (B) Effect of ethanol addition or phosphate removal on swarming in WT, *erdR*, and *phoB* strains on mPGM swarm agar. (C) Heat map of transcripts for 17 genes in the ethanol oxidation cluster in a 12h swarm of *erdR* and PA14 strains. (D) qRT-PCR analysis of 4 genes from the ethanol oxidation cluster and *actP* in a 12h swarm of *erdR* over a 12h swarm of PA14. (E) Swarming of WT, *erdR*, and *erdR*^+^ strains on mPGM swarming agar with 0.1% ethanol. An unpaired *t* test was used for analysis of significance (*, *P* ≤ 0.05; **, *P* ≤ 0.01).

In our previous study of P. aeruginosa swarming on four distinct swarming agar media—PGM, M8, M9, and BM2—we reported that as many as 44 two-component-system (TCS) genes are required for P. aeruginosa swarming in a medium-specific manner (10). We found that six TCS genes were necessary for swarming exclusively on PGM agar while they were dispensable on the other three media. Interestingly, two of the PGM-specific response regulators (EraR and ErdR) are known to be involved in regulating ethanol oxidation in P. aeruginosa (19). Consistent with this observation, we found that the addition of 0.1% ethanol did not affect wild-type (WT) swarming on M9 swarm agar and had slightly reduced swarming in M8 swarm medium (Fig. 2A). It is important to note that Lewis et al. reported that 1% ethanol blocks swarming on M8 swarm agar and flagellar motility (25), suggesting that ethanol has dosage effects on the motility of P. aeruginosa in a medium-dependent manner. We created a mutant with a deletion of *erdR* and found that the mutant was defective in swarming on PGM swarm agar but not on M8 or M9 swarm agar (Fig. S5), consistent with no effect of low concentration ethanol on swarming in M8 and M9 medium. We found that the *erdR* mutant had no growth defect in modified PGM (mPGM) or M9 broth (Fig. S6). As a control, we performed phosphate starvation to induce swarming in PA14 on mPGM agar. As shown in Fig. 2B, either ethanol addition or phosphate removal induces swarming in the WT. The phosphate-related response regulator encoding gene, *phoB*, was required for phosphate starvation-induced swarming but not for the ethanol-induced swarming. In contrast, the ErdR response regulator was needed in the ethanol-induced swarming but was entirely dispensable for phosphate starvation-induced swarming (Fig. 2B). These experiments validated the contextual requirement of response regulators and hence the regulation of swarming in P. aeruginosa.

10.1128/mBio.02033-21.5 FIG S5 (A) Swarming phenotype of the *erdR* mutant on mPGM-EtOH and on M8 and M9 swarm agar. Swarming phenotype of (B) the WT and the (C) *pqqE*, (D) *pqqC*, (E) *pqqB*, (F) *exaC*, (G) *exaA*, (H) *eraR*, (I) *pqqF*, (J) PA14_38950, and (K) PA14_38990 mutants on M9 swarm agar. Download FIG S5, PDF file, 0.2 MB. Copyright © 2021 Badal et al. 2021 Badal et al. https://creativecommons.org/licenses/by/4.0/ This content is distributed under the terms of the Creative Commons Attribution 4.0 International license.

10.1128/mBio.02033-21.6 FIG S6 Analysis of growth of P. aeruginosa PA14 and *erdR* in mPGM broth and M9 broth at 37°C. Download FIG S6, PDF file, 0.1 MB. Copyright © 2021 Badal et al. 2021 Badal et al. https://creativecommons.org/licenses/by/4.0/ This content is distributed under the terms of the Creative Commons Attribution 4.0 International license.

To confirm whether ErdR was indeed necessary for ethanol oxidation-induced swarming, we performed RNA-seq analysis of the *erdR* population in the swarm lag at 12 h compared to the WT in the swarm lag at 12 h. As seen in the RNA-seq analysis (Table S1B), 121 genes were upregulated, and 73 genes were downregulated in the *erdR* mutant compared to the WT. As shown in the heat map, the majority of genes in the 17-gene cluster linked to ethanol oxidation were expressed at lower levels in the mutant (Fig. 2C). Thus, we propose to annotate this cluster as the ErdR regulon. This was further confirmed by qRT-PCR analysis (Fig. 2D). We found that transcript levels of all four genes in the cluster tested (*pqqA*, *exaB*, *exaA*, and PA14_38880) and acetate permease *actP* were lower in the *erdR* population at 12 h in the swarm lag than in the corresponding WT population. This suggested that ErdR is necessary for the transcription of genes involved in the utilization of ethanol. We looked at the ethanol dose response of the *erdR* mutant (Fig. S3F) and found that it was unresponsive to the low concentrations of ethanol tested. Although tendril formation was initiated at 1% ethanol in the *erdR* mutant, the tendrils failed to extend and grow. As expected, complementation of the *erdR* deletion mutant with a wild-type copy of the gene (*erdR*^+^ strain) rescued the swarming phenotype of the mutant on mPGM with ethanol (Fig. 2E). Consistent with the dispensability of ErdR function on M9 swarm agar (Fig. S5A), *pqqB*, *pqqC*, *pqqD*, *pqqE*, *pqqF*, *exaC*, *exaA*, *eraR*, PA14_38950, and PA14_38990 mutants also had no swarming motility defect on M9 swarm agar (Fig. S5B to K).

In all, our results indicated that ErdR response regulator controls ExaA expression and the conversion of ethanol to acetaldehyde necessary for P. aeruginosa swarming in response to ethanol.

### Ethanol is a signal for tendril formation in P. aeruginosa PA14.

**FIG 3:**
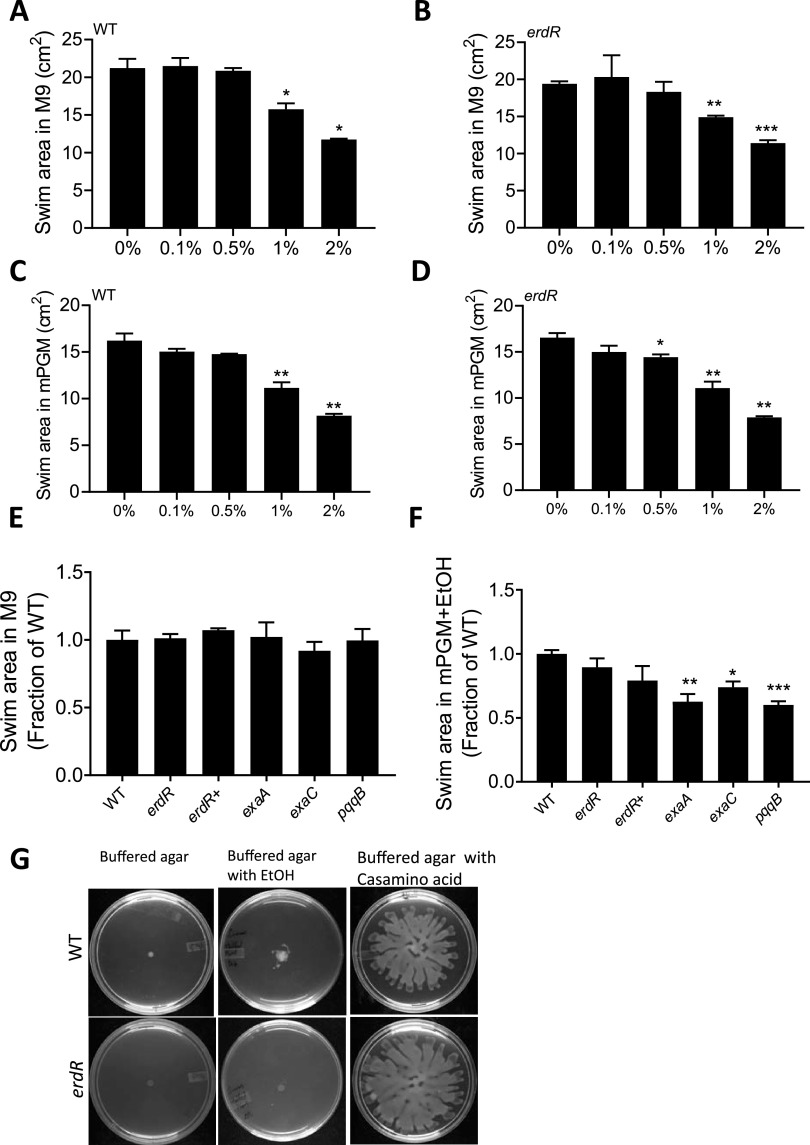
Low-concentration ethanol induces swimming and tendril formation in P. aeruginosa. Effect of different concentrations of ethanol on swimming motility of WT and *erdR* in 0.3% agar in M9 (A and B) and mPGM (C and D) media. Swimming area of *erdR*, *erdR^+^*, *exaA*, *exaC*, and *pqqB* strains in M9 (E) and mPGM (F) swimming agar with ethanol. (G) Effect of ethanol on tendril formation in WT and *erdR* strains on buffered swarm agar without ethanol, with ethanol, or with 0.5% Casamino Acids. An unpaired *t* test with Welch’s correction was used for analysis of significance (*, *P* ≤ 0.05; **, *P* ≤ 0.01; ***, *P* ≤ 0.001).

Lack of induction of swarming with acetate supplementation suggested that ethanol is a signal for swarming in bacteria instead of a carbon source. The signal can induce tendril formation or regulate swimming motility, both conserved features of swarming. We first performed plate-based quantitative swimming assays on 0.3% agar in M9 or mPGM with increasing ethanol concentrations for the WT and the *erdR* mutant. We found that lower concentrations of ethanol had no effect on swimming, but ethanol concentration higher than 1% reduced swimming area in both M9 and mPGM with 0.3% swimming agar (Fig. 3A to D), consistent with a recent report of an effect of 1% ethanol on flagellar stators (25). We found that the swimming motility of *erdR*, *exaA*, *exaC*, and *pqqB* mutants was comparable to that of the WT on M9 swimming medium (Fig. 3E); however, the swimming ability of the *exaA*, *exaC*, and *pqqB* mutants in mPGM was slightly lower than that of the WT on mPGM swimming medium with 0.1% ethanol (Fig. 3F).

We reasoned that low concentrations of ethanol might induce coordinated motility, resulting in tendril formation, a conserved feature of PA14 swarming. To test this, we spotted WT and *erdR* cells on buffered swarm agar plates. These plates lack peptone but contain 20 mM ammonium chloride and either 0.1% ethanol or 0.5% casamino acids. As shown in Fig. 3G, there was no swarming in either the WT or the *erdR* mutant in buffered agar plates. Although the WT and *erdR* strains grew poorly on buffered agar plates where peptone was replaced with 0.1% ethanol, the WT colony could initiate 3 to 5 tendril-like structures, but the *erdR* mutant failed to form tendrils. *erdR* mutants were also much reduced in growth on buffered agar plates with ethanol, consistent with their requirement for the use of ethanol as the sole carbon source (19). These experiments suggested that low-concentration ethanol induces coordinated motility (tendril formation) in bacteria starved for a carbon source. To test if other nutrients can also drive tendril formation and extension, we added 0.5% (wt/vol) casamino acids in buffered agar plates. We found that casamino acids could induce swarm formation, but it was independent of ErdR (Fig. 3G). In all, our study suggested that ErdR-dependent ethanol oxidation is necessary to induce tendril formation in starving bacteria when ethanol is the signal for swarming.

### Ethanol induces swarming motility in starving bacteria.

**FIG 4:**
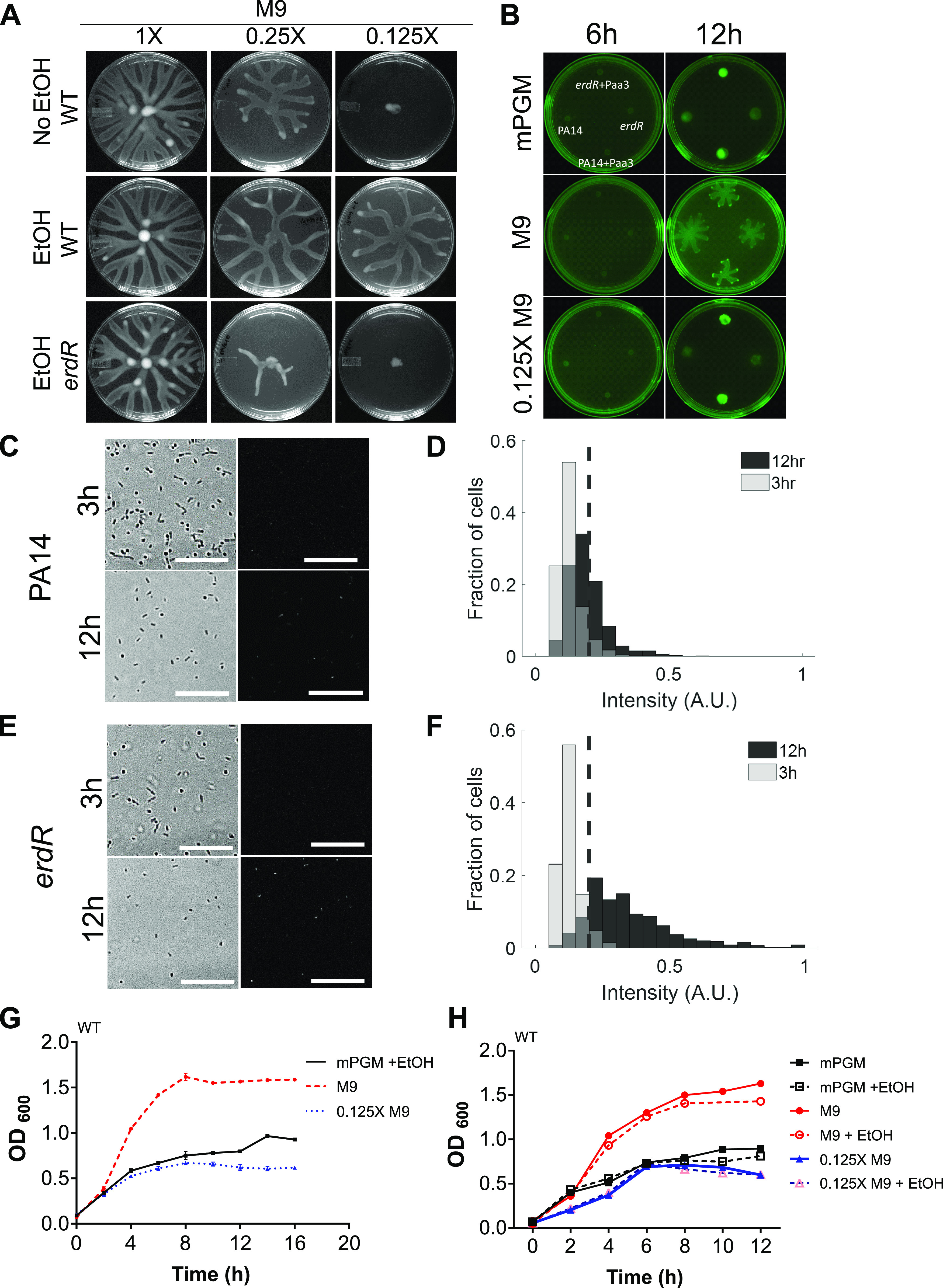
Ethanol induces swarming motility in starving P. aeruginosa. (A) Analyses of swarming of the WT on M9, 0.25× M9, and 0.125× M9 swarm agar with and without ethanol and of the *erdR* mutant with ethanol. (B) Expression of the RpoS reporter P*aa3*::GFP in WT and *erdR* strains on mPGM, M9, and 0.125× M9 swarming agar. Reporterless strains were also included as indicated. Plates had no ethanol, and they were imaged at 6 h and 12 h after spotting. Single-cell imaging was performed 3 h and 12 h after spotting on mPGM–0.1% EtOH for (C) PA14 with P*aa3*::GFP, and its analyzed intensities were plotted as a histogram (D) with light gray for 3-h-old and black for 12-h-old cells; the overlap region is represented by the dark gray color. Similarly, the *erdR* mutant with P*aa3*::GFP was imaged (E), and intensity was analyzed and represented by a histogram (F), with light gray for 3-h-old and black for 12-h-old cells; the overlap region is represented by the dark gray color. The dashed line (in D and F) represents the average background intensity of fluorescence images. (G) Analysis of planktonic growth of WT P. aeruginosa in mPGM broth, M9 broth, and 0.125× M9 broth. (H) Effect of 0.1% ethanol addition on WT growth in mPGM, M9, and 0.125× M9 broth.

Ethanol-induced formation of tendrils on swarm agar without a carbon source was consistent with our previous observation that P. aeruginosa PA14 does not show swarming on rich media such as brain heart infusion (BHI) and LB media (10). Although PA14 swarms well on M9 or mPGM swarm agar with ethanol, its growth is better supported in M9 broth than in mPGM broth, raising the possibility that PA14 cells face nutrient restriction much earlier in mPGM. To test this, we compared WT growth in mPGM and M9 broths. In mPGM broth, WT bacteria entered the stationary phase at an optical density at 600 nm (OD_600_) of ∼0.6, while in M9 broth, the bacteria entered the stationary phase at an OD_600_ above 1.5 (Fig. 4G). We then reduced casamino acids and glucose concentrations in M9 broth to one-fourth (0.25×) and one-eighth (0.125×) of the normal amount used in M9 (1×) while keeping the concentration of divalent cations and phosphate unaltered. Although PA14 swarmed well in 1× M9 swarm agar, it swarmed weakly on 0.25× M9 swarm agar and could not swarm at all on 0.125× M9 swarm agar (Fig. 4A). Interestingly, while the growth rate of PA14 in 0.125× M9 broth was much lower than that in M9 broth, it was comparable to the growth rate in mPGM broth (Fig. 4G). We inferred that the bacteria on 0.125× M9 swarm agar were as nutrient restricted or starved as they were on mPGM swarm agar. We reasoned that only starving bacteria might be primed to respond to the ethanol signal. As we expected, supplementation of ethanol to 0.125× M9 swarm agar induced swarming in PA14 (Fig. 4A). This indicated that ethanol serves as a swarming signal for bacterial populations exclusively under carbon/nitrogen-limiting conditions. To further confirm this, we also examined the swarming phenotype of the *erdR* mutant on 1×, 0.25×, and 0.125× M9 swarm agar and found that *erdR* was necessary for swarming only on 0.125× M9 and partially on 0.25× M9 (Fig. 4A) but it displayed a WT phenotype on M9 swarm agar. We found that RhlR was also necessary for swarming on swarm surfaces (M9, M9 plus ethanol, 0.125× M9, and 0.125× M9 plus ethanol) as expected. However, ErdR was necessary for swarming only on 0.125× M9–ethanol swarm agar plates (Movie S1A to D). To further rule out that ethanol is not used as a carbon source, we tested the effect of ethanol on PA14 growth in broth cultures. As shown in Fig. 4H, ethanol did not alter the growth rate of WT bacteria in PGM, M9, or 0.125× M9 significantly, suggesting that ethanol is not used as an energy source when peptone (in mPGM) or casamino acids and glucose (in M9) are present but serves a signaling function to promote swarming.

10.1128/mBio.02033-21.9 MOVIE S1 Swarming motility of PA14, *erdR*, and *rhlR* strains. PA14 was spotted at the 12 o’clock position, *erdR* strain at 4 o’clock, and *rhlR* strain at 8 o’clock on 90-mm swarm agar plates containing 1× M9 PGM swarm agar with 0.1% ethanol (A), 1× M9 swarm agar without ethanol (B), 0.125× M9 swarm agar without ethanol (C), and 0.125× M9 swarm agar with 0.1% ethanol (D). Download Movie S1, AVI file, 10.1 MB. Copyright © 2021 Badal et al. 2021 Badal et al. https://creativecommons.org/licenses/by/4.0/ This content is distributed under the terms of the Creative Commons Attribution 4.0 International license.

The entry of bacteria into the stationary phase is accompanied by the activation of the stationary-phase sigma factor RpoS (27, 28). To test whether PA14 cells are indeed in the stationary phase toward the end of swarm lag, we utilized a transcriptional green fluorescent protein (GFP) reporter for a P. aeruginosa terminal oxidase gene, *cox* (also called aa3), a reporter for RpoS activity (29). As shown in Fig. 4B, a 12 h-old colony of P. aeruginosa on mPGM agar showed strong induction of GFP expression reflecting its entry into the stationary phase. We found that the *erdR* population also had higher expression of the RpoS reporter at 12 h than at 6 h (Fig. 4B). This suggested that the stationary-phase sigma factor RpoS was active in both the PA14 and *erdR* populations before the end of swarm lag and formation of tendrils. We also tested RpoS reporter expression on M9 and 0.125× M9 and found that GFP expression in WT and *erdR* populations on 0.125× M9 was higher than populations without GFP plasmid, while there was some reporter expression in small populations of cells at the tips of growing tendrils on M9 swarm agar. For further confirmation of RpoS activation in WT and *erdR* population on swarming plates, we analyzed GFP expression in cells of the swarm at 3 and 12 h (Fig. 4C and E). Both WT and *erdR* populations showed increases in reporter expression at the single-cell level with time (Fig. 4D and F) Taken together, our data suggested that P. aeruginosa cells in low-nutrient media have higher RpoS activity and that they respond to ethanol signal. This also indicated that low-concentration ethanol could be a foraging signal for starving bacterial populations.

### Cryptococcus neoformans induces rhamnolipid- and ErdR-dependent foraging motility in P. aeruginosa.

The induction of swarming in starving bacteria by ethanol prompted us to hypothesize that microbes that produce low levels of ethanol might induce chemotactic motility and swarming in P. aeruginosa. They may also serve as a source of nutrition/food for P. aeruginosa, which is consistent with the ability of P. aeruginosa to grow efficiently in cocultures with Staphylococcus aureus, Candida albicans, Cryptococcus neoformans, etc. (30–32). The presence of microbial species such as S. aureus in the vicinity can influence the motility characteristics of P. aeruginosa, as shown recently (32). We were mindful that several microbes can produce ethanol, but they may not be ecologically relevant for P. aeruginosa. We therefore sought to study organisms known to interact with P. aeruginosa or known to share their habitat with P. aeruginosa, such as the human lung. C. neoformans is a pathogenic yeast that inhabits the lungs of immunocompromised individuals (33). P. aeruginosa interaction with C. neoformans requires cell-cell contact and production of virulence factors like phenazines and exotoxins (30, 31).

**FIG 5:**
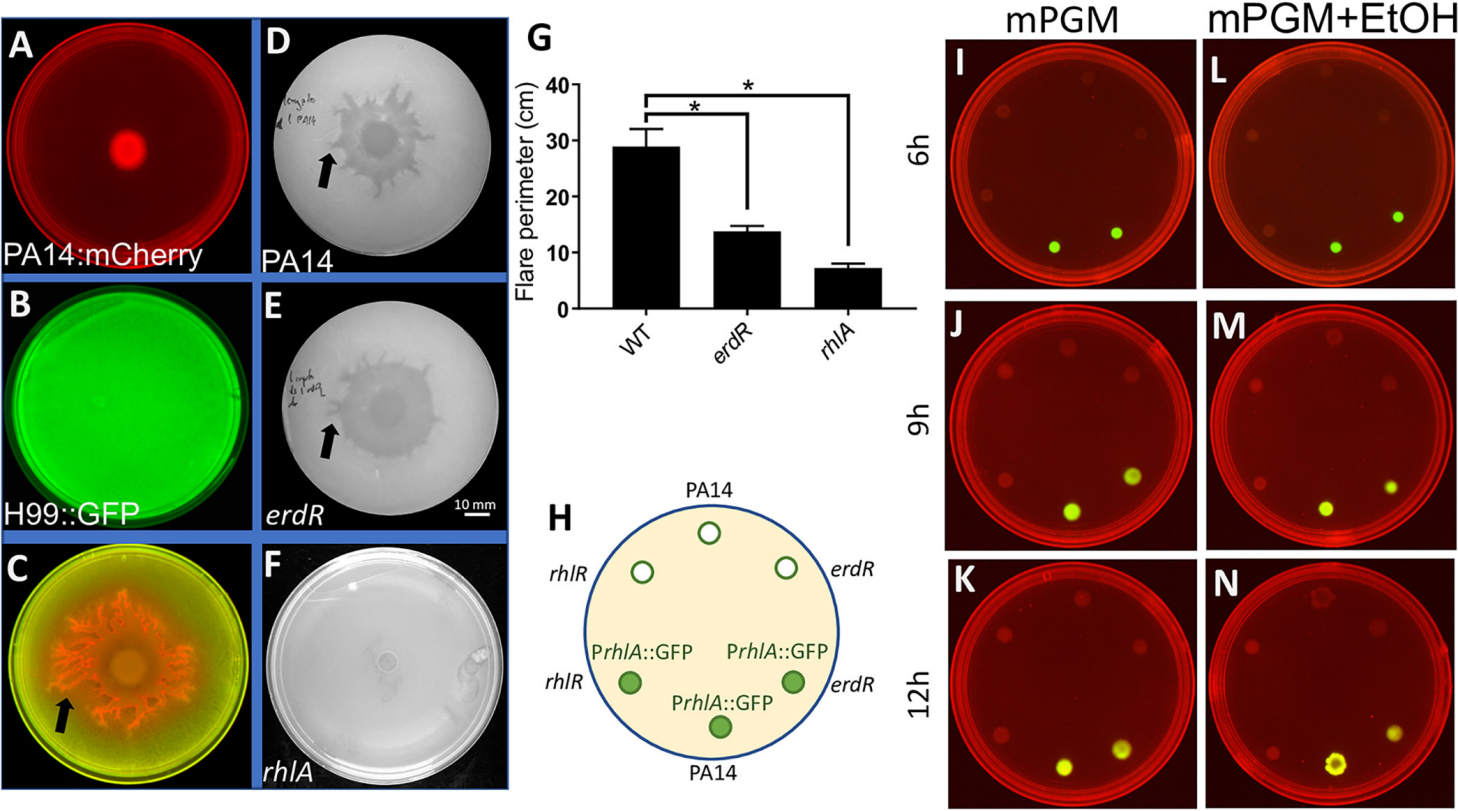
Cryptococcus neoformans induces ErdR-dependent foraging motility in P. aeruginosa. (A) P. aeruginosa PA14::mCherry spotted on BHI–1% agar. (B) Lawn of C. neoformans H99::GFP on BHI–1% agar. (C) P. aeruginosa PA14::mCherry spotted on a lawn of H99::GFP. (D) WT, (E) *erdR* strain, and (F) *rhlA* strain of P. aeruginosa spotted on an H99 lawn. Arrows indicate flares (C, D, and E). (G) Colony perimeter for WT, *erdR*, and *rhlA* strains formed on an H99 lawn. All the plates were incubated at 25°C for 48 h before imaging. An unpaired *t* test with Welch’s correction was used for analysis of significance (*, *P* ≤ 0.05). (H) Schematic of spotting of reporter and reporterless strains on swarm medium plates. (I to N) Expression of the rhamnolipid reporter P*rhlA*::GFP in WT, *erdR*, and *rhlR* strains on mPGM swarm plates with and without ethanol. Reporter expression was assessed at 3, 9, and 12 h after spotting.

We established a plate-based C. neoformans-P. aeruginosa interaction assay by first spreading C. neoformans strain H99 on the entire plate (Fig. 5B) followed by spotting a small (5 μl) volume of P. aeruginosa. We found that P. aeruginosa expressing mCherry grew rapidly over the lawn of H99 expressing GFP and formed broad, tapering tendril-like structures we have termed ‘flares’ (Fig. 5C). On the other hand, WT P. aeruginosa spotted on 1% agar alone (without the yeast) exhibited limited growth and no flares (Fig. 5A), suggesting that C. neoformans induces flare formation and spreading motility in P. aeruginosa colonies. To test whether H99-induced flare formation was dependent on ethanol oxidation, we spotted reporterless WT (Fig. 5D) and *erdR* (Fig. 5E) cells separately on H99 lawns and found that the *erdR* strain formed fewer flares (Fig. 5G). Interestingly, we found that RhlA, essential for rhamnolipid surfactant and swarming in P. aeruginosa, was also essential for colony growth and flare formation by P. aeruginosa on C. neoformans lawns (Fig. 5F and G). This suggested that rhamnolipids, recognized as essential drivers of swarming motility in P. aeruginosa, are also essential for flare formation and spreading of P. aeruginosa on surfaces covered with yeast. We found that an *rhlR* mutant was also defective in flare formation as expected (data not shown). We also analyzed the swarming phenotype of rhamnolipid deficient (*rhlA* deletion) mutant and found that it was completely defective in swarming on mPGM agar with 0.1% ethanol (Fig. S7). We examined *rhlA*::GFP reporter expression in WT, *erdR*, and *rhlR* strains on mPGM swarm agar with and without ethanol (Fig. 5H). The *rhlR* mutant was defective in reporter expression at all time points (6 h, 9 h, and 12 h after spotting), while reporter expression increased with time in the WT, as expected (Fig. 5I to N). The *erdR* mutant had normal basal expression of the reporter at 3 h, but it had low reporter expression at 9 and 12 h compared to WT at the same time points (Fig. 5I to N).

10.1128/mBio.02033-21.7 FIG S7 Swarming of *rhlA* and WT strains of P. aeruginosa on mPGM swarm agar plates with 0.1% ethanol. Imaging was done after 24 h of incubation at 37°C. Download FIG S7, PDF file, 0.1 MB. Copyright © 2021 Badal et al. 2021 Badal et al. https://creativecommons.org/licenses/by/4.0/ This content is distributed under the terms of the Creative Commons Attribution 4.0 International license.

Taken together, our analyses of these spreading behaviors of P. aeruginosa colonies on ethanol-producing yeast indicated that rhamnolipids are essential for the flare formation and spread of P. aeruginosa on yeast cells, while ErdR also contributes to the spread underlying foraging motility.

### Ethanol as a volatile is effective in inducing swarming in P. aeruginosa.

**FIG 6:**
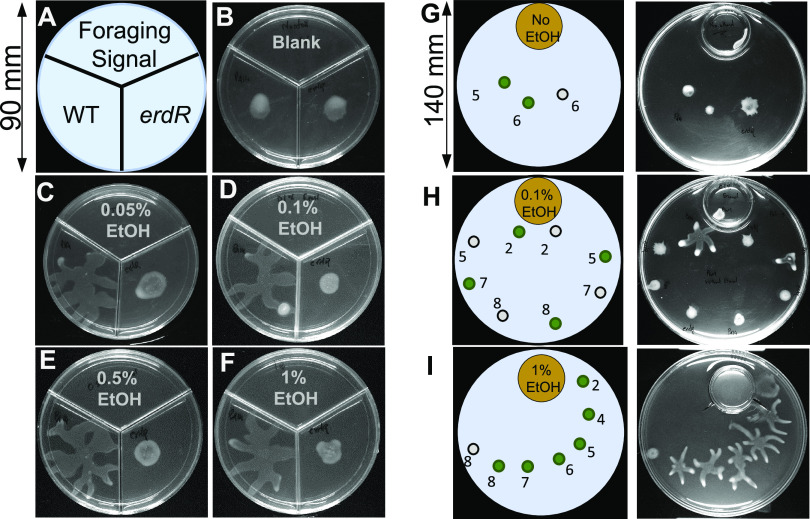
Ethanol in its volatile form induces swarming in P. aeruginosa. (A) Schematic of ethanol delivery as a volatile. (B to F) Effect of various concentrations of volatile ethanol on swarming of WT and *erdR*. (G to I) Schematic of ethanol delivery in a large 140-mm area and effect of 0.1% and 1% ethanol on swarming in PA14 and *erdR* strains on PGM swarm agar lacking ethanol. Gray spots indicate *erdR* populations, and green spots indicate WT populations. Numbers represent the minimum distance, in centimeters, from the edge of plate containing ethanol.

In nature, bacteria face starvation often, and their ability to sense and move toward spatially separated sources of nutrition can be a great survival strategy in a competitive environment. To test whether ethanol could work as a volatile foraging signal, we separated P. aeruginosa cells on swarm media from ethanol by a nonpermeable polystyrene barrier in a tripartite plate (see the schematic in Fig. 6A). In each of the two sections of a tripartite plate (containing mPGM swarm agar), we separately spotted the WT and the *erdR* mutant, while the third section contained mPGM agar without ethanol or with various concentrations of ethanol as a foraging signal. We found that the presence of ethanol in the third section (2 cm away from P. aeruginosa spot) induced swarming in PA14 cells (Fig. 6B to F) at all concentrations tested, but not in *erdR* cells. We also analyzed if methanol, isopropanol, acetate, and acetaldehyde can induce swarming as wafting volatiles but found no swarming (Fig. S3F).

To test the spatial limit of the action of ethanol as a foraging signal, we separated P. aeruginosa cells from the source of ethanol in a larger area (140 mm polystyrene plate). The foraging signal was placed in a 35 mm dish (see the schematic in Fig. 6G–I), while P. aeruginosa was spotted at four distances (2 cm, 5 cm, 7 cm, and 8 cm) from the source of 0.1% ethanol. We found that swarming was induced in WT cells up to 5 cm distance in an ErdR dependent manner (Fig. 6G and H). When we increased the ethanol concentration in the 35 mm dish to 1%, we saw swarming in the WT population as far away as 8 cm (Fig. 6I). Thus, these results showed that ethanol wafting from a distance as great as 8 cm can induce swarming in P. aeruginosa, suggesting that ethanol can mediate the spread of P. aeruginosa via induction of swarming.

### Ethanol signal induces swarming in stalled populations of P. aeruginosa.

**FIG 7:**
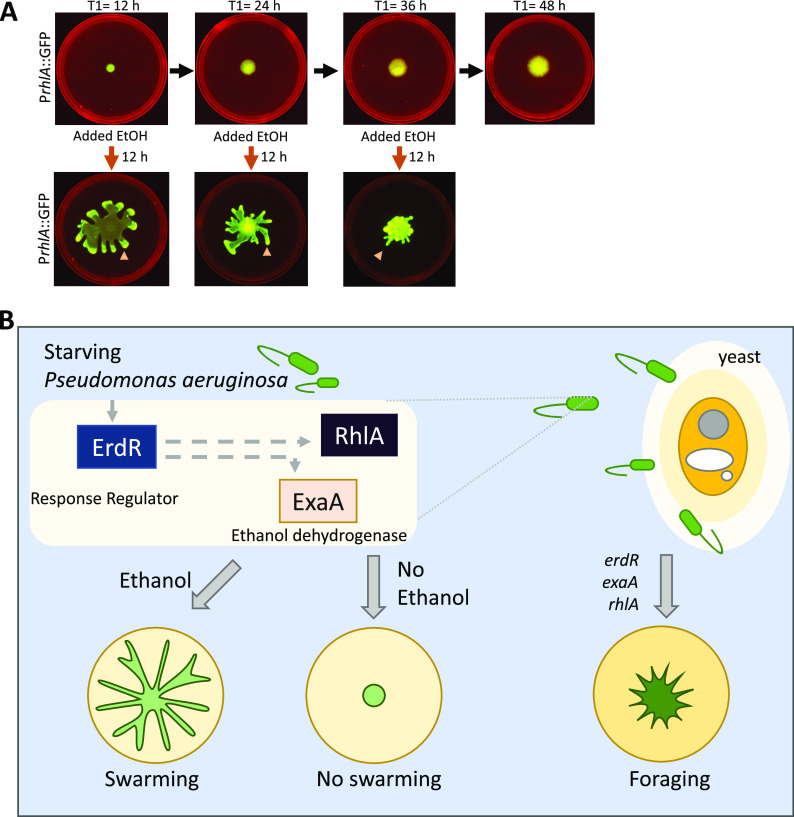
Ethanol can induce swarming in stalled population of P. aeruginosa. (A) Ethanol can induce swarming in populations of bacteria stalled on a mPGM swarm agar surface for 12, 24, and 36 h. Induction of the rhamnolipid reporter P*rhlA*::GFP in tendrils formed after addition of ethanol is indicated with arrows. (B) Model describing the effect of low-concentration ethanol on P. aeruginosa. Ethanol can induce swarming in starving bacteria on a swarm surface in a manner which is dependent on the response regulator ErdR required for upregulation of ExaA, enzyme for the oxidation of ethanol, and rhamnolipids. ErdR also regulates the foraging motility of P. aeruginosa on a lawn of the ethanol-producing yeast Cryptococcus neoformans.

Bacteria often encounter starvation causing stasis effects on their growth. We wondered if starving cells on the swarm surface can retain their ability to swarm and for how long. To test this, we utilized WT P. aeruginosa expressing GFP under the control of the *rhlA* promoter. We spotted the bacteria on mPGM swarm agar and imaged plates at different time points. We incubated these plates without ethanol for 12, 24, and 36 h and found that colony growth was stalled as circular colonies without tendrils (Fig. 7A). We added 0.1% ethanol in the periphery of the plate with stalled colonies and allowed growth for the next 12 h. As shown in Fig. 7A, all the stalled populations responded to ethanol by forming tendrils and grew as a swarm, while one without ethanol remained stalled (Movie S2). These results indicated that cells on a swarm surface could stay in a primed, swarming-proficient state for up to 36 h. This result also showed that ethanol is a powerful motivator of swarming in P. aeruginosa.

10.1128/mBio.02033-21.10 MOVIE S2 Induction of swarming in stalled P. aeruginosa populations upon exposure to ethanol on mPGM swarm agar. (A) P. aeruginosa on mPGM without ethanol; (B) P. aeruginosa stalled on an mPGM plate for 12 h followed by ethanol exposure; (C) P. aeruginosa stalled on a mPGM plate for 24 h followed by ethanol exposure. Total duration of the movie was 50 h for all 3 plates. Download Movie S2, AVI file, 3.8 MB. Copyright © 2021 Badal et al. 2021 Badal et al. https://creativecommons.org/licenses/by/4.0/ This content is distributed under the terms of the Creative Commons Attribution 4.0 International license.

We propose a model (Fig. 7B) to explain the effect of ethanol as a signal for swarming. Low-concentration ethanol can induce swarming in P. aeruginosa population under nutrient-limiting conditions. This is dependent on the ErdR response regulator necessary for the oxidation of ethanol as well as on rhamnolipid surfactant. ErdR also regulates the foraging motility of P. aeruginosa on a lawn of the ethanol-producing yeast C. neoformans.

## DISCUSSION

The work presented here primarily examined specific environmental signals that induce swarming in P. aeruginosa, with an ecological perspective. Our analyses of the transcriptome of bacteria in the early and late phases of swarm lag uncovered the critical role of the periplasmic ethanol oxidation machinery controlled by the response regulator ErdR in P. aeruginosa swarming. Our observation of a swarming defect in ethanol oxidation mutants, involving the alcohol dehydrogenase ExaA or its cofactor (PQQ), further established the critical role of ethanol oxidation in PA14 swarming. Subsequently, we showed that exogenous ethanol imparts its swarm-inducing effects at low concentrations in nutritionally limiting medium, PGM or M9 medium, diluted for carbon and nitrogen. Using a starvation reporter, we showed that ethanol is an environmental trigger for swarming motility in P. aeruginosa, but only in a nutrient-limitation context.

Higher carbon-to-nitrogen ratios are conducive to rhamnolipid production in P. aeruginosa (34, 35). Could ethanol promote swarming by altering this ratio in favor of carbon? Our experiment with an acetaldehyde dehydrogenase mutant or with acetate supplementation suggested that this is unlikely to be the case. We examined whether the ethanol-induced modulation of swarming motility in P. aeruginosa could have arisen in an ecological context. Ethanol-producing microbes are abundant in nature. Of these, we chose C. neoformans, a lung-inhabiting pathogenic yeast (23, 33), to study its interaction with P. aeruginosa. In a coculture experiment of PA14 with C. neoformans, we observed that PA14 displayed robust foraging motility in an ErdR-dependent manner. The flares produced by P. aeruginosa appear similar to tendrils in a swarm in terms of the extension property and their dependence (although partial) on the response regulator ErdR and on rhamnolipid surfactants.

We made a striking observation that ethanol, as a volatile, could induce swarming in PA14 from a distance as great as 8 cm. Also, ethanol could induce swarming in the PA14 population stalled for a long duration, up to 36 h. This suggested that the P. aeruginosa population can exist in nature, in the environment, or in and around its host for a long time in a swarming-proficient, primed state, waiting for foraging signals to allow them to spread. The idea that P. aeruginosa has evolved to perceive a low concentration of ethanol as a foraging signal may appear to contradict the observation that higher concentrations (10- to 20-fold) of ethanol have a negative impact on swimming motility (shown by Lewis et al. [25] and on M9 and mPGM in this study). One possible explanation of this could be that while a lower concentration of ethanol promotes swarming in bacteria, reaching higher concentrations (closer to the microbe producing ethanol) causes slowing down so that P. aeruginosa could dwell there and feed on the microbes.

Environmental cues are known to regulate collective behaviors of bacteria. For instance, phosphate-limitation can stimulate swarming in P. aeruginosa in a manner that relies on the PhoB/PhoR two-component system (8). Similarly, the lack of iron in the swarm medium can promote swarming (9). A recent report suggests a link between ethanol and phosphate signaling in the antagonistic interaction between P. aeruginosa and Candida albicans (36). Our results confirm the requirement for *phoB* under phosphate limitation but also clearly establishes its dispensability during ethanol-stimulated swarming. Conversely, ErdR is not required for phosphate starvation-induced swarming but is essential for ethanol-induced swarming, indicating that P. aeruginosa utilizes independent perception pathways to respond to specific environmental cues. Interestingly, conversion of ethanol to acetaldehyde is necessary for swarming in P. aeruginosa, suggesting that the bacterium has evolved to utilize a periplasmic enzyme to produce a second signal, acetaldehyde, which can likely be compartmentalized in the periplasm for efficient signaling. Other bacteria lack periplasmic alcohol dehydrogenase suggesting that they may not respond to ethanol as swarming signal.

The presence of ethanol response mechanisms partly explains the nutrition-dependent plasticity in swarm characteristics of P. aeruginosa we reported recently (10). It is also reported that the ethanol dehydrogenase ExaA shares strong structural similarity with methanol dehydrogenase found in some other bacteria, such as Methylobacterium extorquens (37). Apart from the structural resemblance, homology between the regulatory components has also been observed (19). The limited swarming we observed with methanol and isopropanol supports this idea. Regardless of differences in the environmental cues, a general principle operating in bacteria such as P. aeruginosa appears to be a population-level coordinated response to starvation. We observed that a small population of bacteria at the tip of the moving tendril expressed the RpoS reporter even on M9 swarming medium (Fig. 4B). As macronutrients, like carbon and nitrogen, and trace elements, such as iron and phosphorus, are essential for growth, the bacterium appears to have invested part of its large sensory repertoire, the TCS, in the search for foraging cues.

The ability of a stalled population to respond to exogenous ethanol suggests several possible scenarios with relevance for its pathogenesis and spread: (i) the bacterium is capable of surviving in a swarming-proficient state for over a day, and (ii) ethanol might provide a chemotaxis signal for the population. The former suggests that starving Pseudomonas is primed to spread faster, which would be crucial in treating P. aeruginosa infection. Our observation also supports the second scenario wherein tendrils are initiated by low-concentration ethanol. Further confirmation comes from the observation that tendril formation is dependent on ErdR. In P. aeruginosa, a unique set of chemotactic systems drives swimming and twitching motilities, and the existence of an additional putative chemotaxis system has also been described (38). Perhaps one of these could be activated by low-concentration ethanol.

Our study provides a broader perspective on the effect of ethanol on P. aeruginosa. Despite the antimicrobial effect of high-concentration ethanol and its repressive effects on flagellar motility, our study has uncovered a positive impact of low-concentration ethanol on swarming, especially under nutrient-limiting conditions, a situation that bacteria might often encounter in nature. The effect of ethanol as a volatile at a distance a million times the body size of the bacterium suggests that the presence of ethanol-producing microbes at reasonably large distances can cause the collective spread of a P. aeruginosa population. Future studies will help decipher mechanisms by which ethanol and other foraging signals are processed in the individual bacterial cell and coordinated with quorum-dependent signals to execute collective motion during swarming.

## MATERIALS AND METHODS

### Microbes and growth conditions.

Pseudomonas aeruginosa PA14 WT and the isogenic transposon-insertion mutant strains (21) were obtained from Frederick Ausubel. The markerless deletion strains (*erdR* and *rhlA*) and *erdR* complemented strain (*erdR^+^*) were generated in this study. Additional strains used in this study are listed in Table S1C. Unless otherwise mentioned, P. aeruginosa WT and markerless deletion strains were routinely cultured (at 37°C) in LB, while the transposon insertion mutants were cultures in LB with 50 μg ml^−1^ gentamicin. Similarly, E. coli strains used for cloning experiments are routinely cultured (at 37°C) in LB with appropriate antibiotic selections, whenever required. Cryptococcus neoformans H99 was cultured in yeast extract-peptone-dextrose (YPD) medium at 25°C. Additional media used in this study include PGM (50 mM NaCl, 0.32% peptone, 2.5% 1 M KPO_4_ buffer [pH 6.0], 1.0 mM MgSO_4_, 1.0 mM CaCl_2_, 5 mg ml^−1^ cholesterol [dissolved in absolute ethanol]). Since we determined (Fig. S2) that solvent ethanol is responsible for the swarm-inducing effect of PGM, PGM without ethanol is referred to as modified PGM (mPGM) (50 mM NaCl, 0.32% peptone, 2.5% 1 M KPO_4_ buffer [pH 6.0], 1.0 mM MgSO_4_, 1.0 mM CaCl_2_). M9 medium is 8.6 mM NaCl, 20 mM NH_4_Cl, 1.0 mM CaCl_2_,1.0 mM MgSO_4_, 22.0 mM KH_2_PO_4_, 12.0 mM Na_2_HPO_4_, 0.2% glucose, and 0.5% Casamino Acids. M8 is a modification of M9 medium (excluding NH_4_Cl and CaCl_2_ salts) (3). Buffered swarm agar (20 mM NH4Cl, 50 mM NaCl, 0.32% peptone, 2.5% 1.0 M KPO_4_ buffer [pH 6.0], 1.0 mM MgSO_4_, 1.0 mM CaCl_2_) is similar to mPGM but lacks peptone.

All planktonic-phase growth assays for P. aeruginosa strains were done at 37°C, and growth was assessed by measuring OD_600_ every 1 to 3 h with two or three biological replicates.

### Motility assays.

Swarming motility assays were performed as described earlier (10). An appropriate medium (PGM, M8, or M9) was solidified with 0.6% Bacto agar (BD) and stored for 16 h at room temperature. All plates were inoculated at the center with 2 μl of overnight bacterial culture in LB broth (OD_600_ = 2.8 to 3.0) and incubated at 37°C for 24 h more. Images of the swarm were captured using the Vilber E-box.

Swimming assays were performed on LB medium containing 0.3% Bacto agar (BD). The secondary culture (OD_600_ = 1) was introduced into the center of the swimming agar plate using a pipette tip by puncturing into the agar but without touching the base of the plate. Plates were incubated at 37°C for 24 h right side up, and images were captured using the Vilber E-box.

### Coculture assay.

In the coculture experiments, WT P. aeruginosa PA14 or mutants (*erdR*, *erdR^+^*, *exaA*, *exaC*, *pqqB*, *rhlR*, or *rhlA*) were individually spotted on a lawn of C. neoformans H99 on a 90-mm plate containing BHI agar 1% (wt/vol). A 500 μl of overnight culture (OD_600_ diluted to 1) of yeast was spread over a freshly prepared BHI plate and allowed to dry for 40 to 50 min. Subsequently, 0.5 μl of P. aeruginosa culture (diluted to an OD_600_ of 1) was spotted at the center of the yeast lawn. Plates were incubated at 25°C for 48 h, followed by imaging using the ChemiDoc system (Bio-Rad).

### Sample preparation for RNA-seq and qRT-PCR analysis.

At 3 h and 12 h after spotting of bacteria on swarm agar plates, cells from swarming colonies (6 colonies per 90-mm PGM swarm agar plate) were harvested using chilled phosphate-buffered saline (PBS) buffer. Total RNA was extracted from a final volume of 2 ml of harvested cells (normalized to an OD_600_ of 1) by the hot-phenol method (39). Briefly, cells chilled on ice were centrifuged at 5,000 rpm for 5 min. The pellet was resuspended in lysis buffer (20 mM Tris-HCl [pH 7.5], 20 mM NaCl, 5 mM Na_2_EDTA, 5 mM vanadyl ribonucleoside complex (VRC), SDS [1%, wt/vol], and 2-mercaptoethanol [0.7%, vol/vol]) to which an equal volume of hot phenol (pH 4.3, 65°C) was added and vortexed, followed by incubation at 65°C for 6 min. Samples were centrifuged at 12,000 rpm for 15 min to recover the aqueous layer, which was further extracted using an equal volume of phenol-chloroform mixture (12,000 rpm, 10 min) followed by an equal volume of chloroform (12,000 rpm, 7 min). The RNA was precipitated with ethanol and resuspended in 0.5 ml of molecular-grade water. Subsequently, RNA purity was analyzed using a spectrophotometer (NanoDrop 1000) and subjected to DNase I treatment. For the RNA-seq experiments, total RNA preparations were further subjected to rRNA depletion with a Ribominus bacterial transcriptome isolation kit (K155004) before library preparation.

### Library preparation and RNA-seq analysis.

The cDNA libraries were prepared from the rRNA-depleted RNA samples using the NEBNext Ultra II directional RNA library preparation kit for Illumina. RNA sequencing was done on the Illumina NextSeq 500 system by Genotypic Technology Pvt. Ltd., Bangalore, India. The raw data were processed using the HISAT2-StringTie pipeline as described elsewhere (40). The reads were mapped to the P. aeruginosa UCBPP-PA14 reference genome (NC_008463.1) using the HISAT2 alignment tool. StringTie was used to assemble reads and generate FPKM (fragments per kilobase per million) values. After filtering undesirable contaminants (such as rRNAs), differentially expressed genes between the samples were identified [the 12-h swarm over the 3-h swarm of the WT and the 12-h swarm of the Δ*erdR* mutant over the 12-h swarm of the WT were determined as fold change (FC) by the formula log_2_(mean FPKM_test_*/*mean FPKM_control_)], with thresholds of adjusted *P* values at ≤0.05 and an absolute fold change of 1.5 or above. The complete list of differentially expressed genes is available in Table S1A and B.

Heat maps were generated using Morpheus (https://software.broadinstitute.org/morpheus), web-based matrix analysis and visualization software from the Broad Institute. In the heat map, the first three columns are the three replicates of the control sample (base) and the last three columns are the three replicates of the test sample. The hierarchical clustering merges objects (here, sample sets) based on their pairwise distance. The closest samples were merged first, while the farthest apart were merged last. The result is a tree dendrogram where the lowest (leaf) nodes represent original samples and internal (higher) nodes represent the merges that occurred. So, in the heat map (Fig. 1B and 2C), the first three columns (which forms a cluster) are replicates for the 3-h swarm lag and the last three columns, which also cluster, are the replicates for the 12-h swarm lag.

### qRT-PCR analysis.

DNase I-treated RNA was quantified on a NanoDrop spectrophotometer (ND-1000). cDNA was prepared using an iScript cDNA synthesis kit (Bio-Rad). The iTaq Universal SYBR green Supermix (Bio-Rad) was used for qRT-PCR using a Quant Studio 3 real-time PCR system (Applied Biosystems). We used four or five biological replicates for each sample and two technical replicates for each biological replicate. Fold change for each transcript was calculated from the cycle threshold (*C_T_*) values normalized to the housekeeping gene *rpoD* following the ΔΔ*C_T_* method (41). Primers used for qRT-PCR are described in Table S1D.

### Construction of markerless deletion strains.

Markerless deletion strains (*erdR* and *rhlA*) were generated by the two-step allelic exchange strategy (42, 43). Briefly, upstream and downstream flanking sequences (∼600 bp) of the target coding sequences were amplified from the PA14 chromosomal DNA using appropriate primer pairs (Table S1D). The fragments were then cloned into the suicide vector pEXG2 (44) to generate the desired deletion construct. The construct was then introduced into PA14 (WT) by conjugation using E. coli SM10 as the donor strain (45). Merodiploids were selected under gentamicin selection followed by growth in selection-free broth for a brief period (∼1 h). Finally, the deletion strains were selected by the sucrose sensitivity counterselection approach. Clones were screened by deletion-specific PCR and confirmed by sequencing.

### Complementation of *erdR*.

Complementation of *erdR* was done as described elsewhere (43). The *erdR* gene, including the promoter, was PCR amplified from the WT chromosomal DNA using the primer pair (Table S1D) and cloned into the mini-CTX-1 vector (46) to generate mini-CTX1-*erdR*. This construct was transformed into E. coli SM10. Using a conjugative transfer approach, the complementation construct was moved from the E. coli SM10 donor to an *erdR* strain of P. aeruginosa. Transformants were selected on LB plates containing 50 μg/ml tetracycline. Complementation was verified by PCR amplification followed by the rescue of the *erdR* phenotype. The complemented strain is represented as *erdR*^+^ here.

### Conjugative transfer of reporter plasmids and reporter assays.

The reporter plasmids pLD2958 (P*aa3*::GFP) (29) and pJF101 (P*rhlA*::GFP) (47) (Table S1C, E) were transformed (48) into the E. coli SM10 donor strain. The plasmids were moved from SM10 to the appropriate P. aeruginosa recipient strain by conjugative transfer (45). Transformants were selected on an LB agar plate containing appropriate antibiotics.

Reporter assays were performed using an overnight culture of P. aeruginosa PA14 strains—WT, *erdR*, and *rhlR* strains—transformed with a GFP reporter. Two microliters of the culture was spotted on appropriate swarming plates. These plates were kept at 37°C and imaged at 475 nm/510 nm (for GFP) and 546 nm/568 nm (for mCherry) using a Bio-Rad ChemiDoc MX system.

### Flare perimeter measurement.

The flare images were imported into the MATLAB (2019b) software. These images were first converted into binary images with the whole plate using a thresholding method. This binary image was then used to estimate the length of a pixel in centimeters (pixel/centimeter ratio) by counting the number of pixels in the diameter of the plate (90 mm) divided by 9 cm. Then we created a binary mask image to segment out the flare from the whole image. We used the bwareaopen and imfill functions of the image processing toolbox to remove any salt-pepper noise in the segmented image. Finally, we estimated the perimeter using the regionprops function and divided it by pixel/centimeter.

### RpoS reporter imaging.

PA14 with a Paa3::GFP reporter plasmid (29) was spotted on a PGM agar plate and incubated at 37°C. A loopful of a bacterial colony was taken from a spotted plate at 3 and 12 h and resuspended in 50 μl PBS buffer on a glass slide. The glass slide was observed under a Leica DMi8 inverted fluorescence microscope at ×40 magnification. Images were captured with bright-field and GFP fluorescence settings. These captured images were then imported into MATLAB (2019b) using the image processing toolbox function imread. These bright-field images were then passed through the adaptive threshold function to remove background from the images of bacteria, followed by identification of the centroids of individual bacteria in an image by using the regionprops function on bright-field images. Using the centroid table, a column of the average fluorescence intensity for each bacterium was created and plotted as an overlapping histogram with the intensity array from the 3-h and 12-h data sets. Further, we took the average background fluorescence intensity and used it to distinguish the expression of GFP from background noise. Average fluorescence intensity of each bacterium was acquired using centroids obtained from brightfield images and plotted as an overlapping histogram for 3- and 12-hours old bacteria spots.

### Data availability.

Fastq files for RNA-seq are available at NCBI (BioProject ID PRJNA608122).
