# Supplementary material for: Foraging Signals Promote Swarming in Starving Pseudomonas aeruginosa
Source: mBio. 2021 Oct 5;12(5):e02033-21. doi: 10.1128/mBio.02033-21 (PMC8546858; doi:10.1128/mBio.02033-21)
Supplement: FIG S1 [file mbio.02033-21-sf001.pdf]

S1

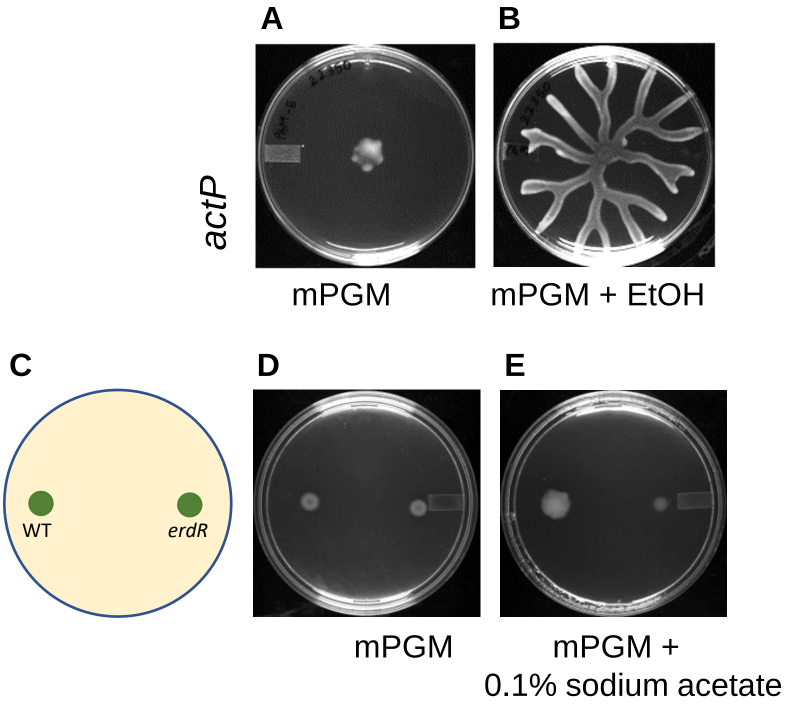

Figure S1: Acetate permease mutant (*actP)* swarming on (A) mPGM agar (B) mPGM agar supplemented with ethanol (0.1% v/v). Effect of acetate supplementation on swarming of WT and *erdR* mutant of *P. aeruginosa* PA14 shown in (C) schematic, (D) on mPGM-0.6% agar and (E) on mPGM-0.6% agar with 0.1% sodium acetate.
