# Supplementary material for: Foraging Signals Promote Swarming in Starving Pseudomonas aeruginosa
Source: mBio. 2021 Oct 5;12(5):e02033-21. doi: 10.1128/mBio.02033-21 (PMC8546858; doi:10.1128/mBio.02033-21)
Supplement: FIG S2 [file mbio.02033-21-sf002.pdf]

S2

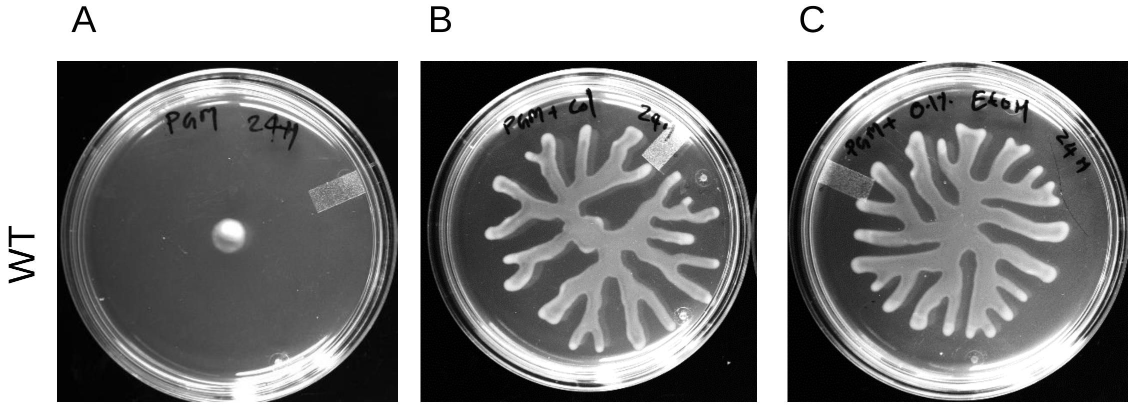

	A	B	C
mPGM	+	+	+
Cholesterol#	-	+	-
Ethanol (0.1%)	-	+	+

Figure S2. Swarming motility of *P. aeruginosa* on swarm agar (A) mPGM (B) mPGM supplemented with cholesterol (#dissolved in 0.1% ethanol) (C) mPGM with 0.1% ethanol.
