# Supplementary material for: Foraging Signals Promote Swarming in Starving Pseudomonas aeruginosa
Source: mBio. 2021 Oct 5;12(5):e02033-21. doi: 10.1128/mBio.02033-21 (PMC8546858; doi:10.1128/mBio.02033-21)
Supplement: FIG S3 [file mbio.02033-21-sf003.pdf]

S3

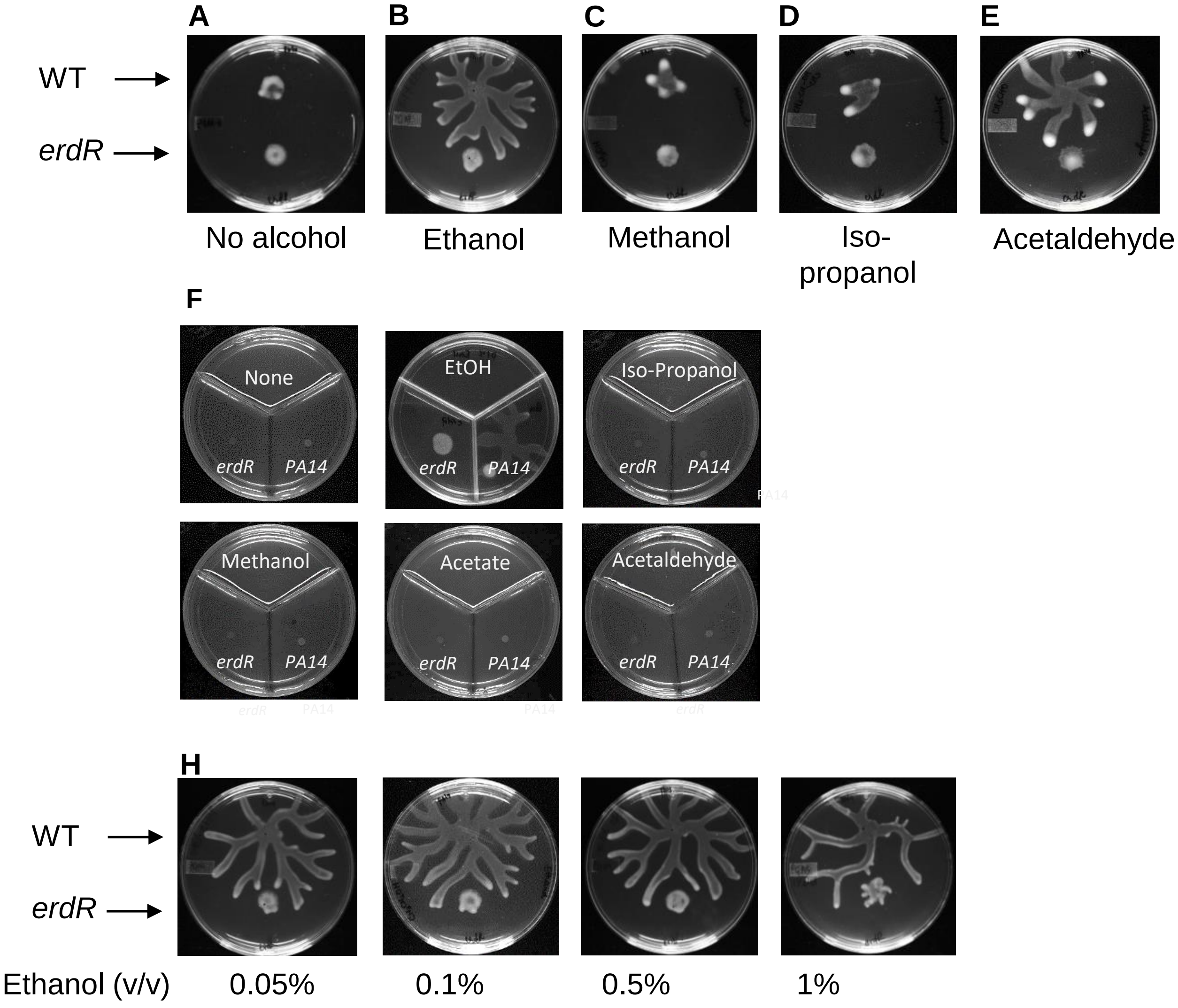

Figure S3: *P. aeruginosa* and *erdR* swarming on (A) mPGM (B) mPGM supplemented with 0.1 % ethanol (C) mPGM supplemented with 0.1% methanol (D) mPGM supplemented with 0.1% iso-propanol (E) mPGM supplemented with 0.1% acetaldehyde. (F) WT and erdR swarming on mPGM supplemented with 0.1% of ethanol, iso-propanol, methanol, sodium acetate and acetaldehyde in trans, in tripartite plates. (G) WT and *erdR* swarming on mPGM supplemented with various concentrations of ethanol.
