# Supplementary material for: Foraging Signals Promote Swarming in Starving Pseudomonas aeruginosa
Source: mBio. 2021 Oct 5;12(5):e02033-21. doi: 10.1128/mBio.02033-21 (PMC8546858; doi:10.1128/mBio.02033-21)
Supplement: FIG S4 [file mbio.02033-21-sf004.pdf]

S4

A B C D

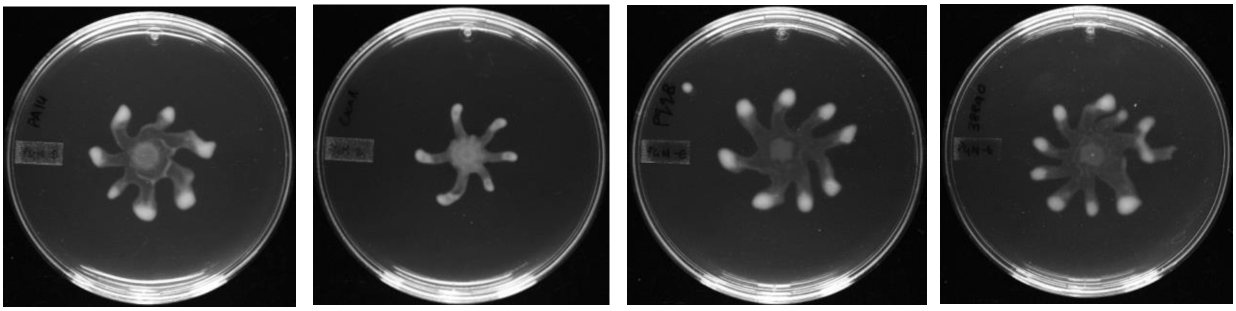

WT *exaA* *pqqB* *exaC*

Figure S4: *P. aeruginosa* swarming on mPGM supplemented with 0.1% acetaldehyde for (A) WT (B) *exaA* (C) *pqqB* (D) *exaC.*
