# Supplementary material for: Foraging Signals Promote Swarming in Starving Pseudomonas aeruginosa
Source: mBio. 2021 Oct 5;12(5):e02033-21. doi: 10.1128/mBio.02033-21 (PMC8546858; doi:10.1128/mBio.02033-21)
Supplement: FIG S5 [file mbio.02033-21-sf005.pdf]

S5

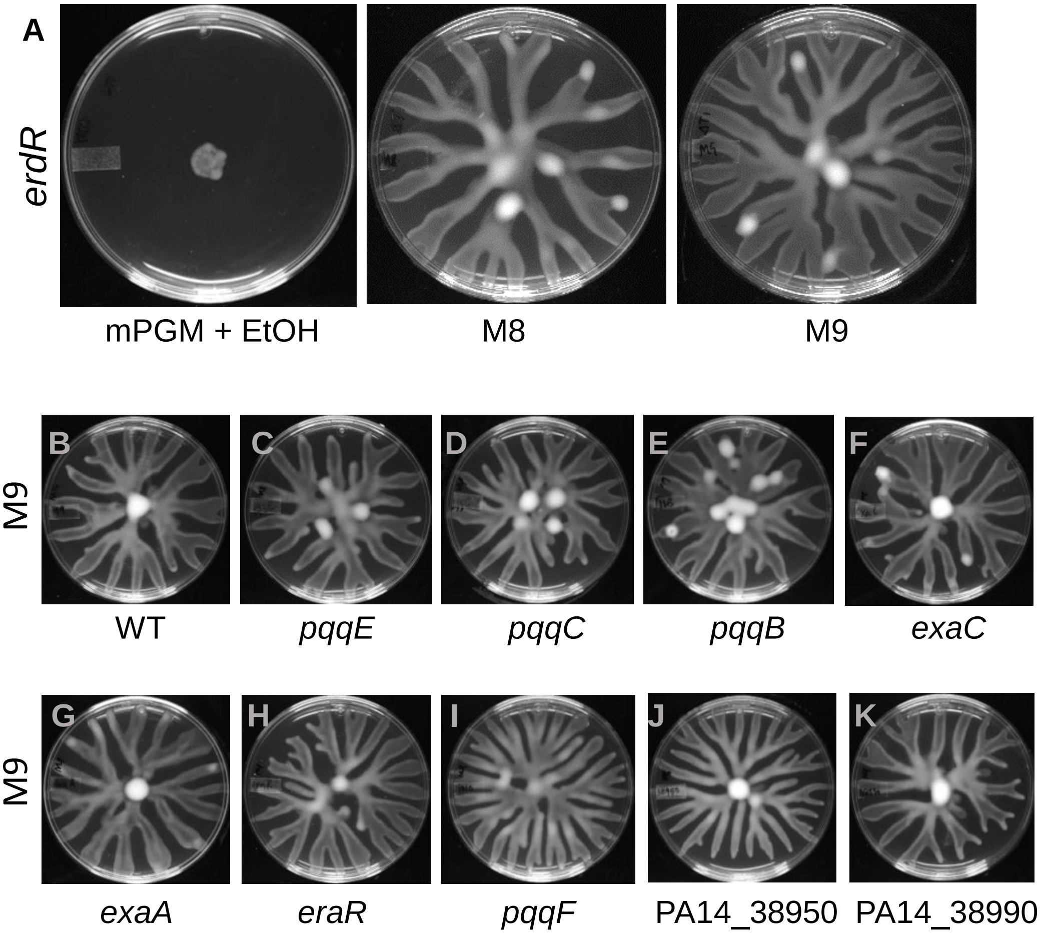

Figure S5: (A) **S**warming phenotype of *erdR* mutant on mPGM+EtOH, on M8 and on M9 swarm agar. Swarming phenotype of (B) WT, (C) pqqE (D) *pqqC,* (E) *pqqB,* (F) *exaC,* (G) *exaA,* (H) *eraR,* (I) *pqqF,* (J) PA14_38950, and (K) PA14_38990 mutants on M9 swarm agar.
