# Supplementary figures and images for: Foraging Signals Promote Swarming in Starving Pseudomonas aeruginosa
Source: mBio. 2021 Oct 5;12(5):e02033-21. doi: 10.1128/mBio.02033-21 (PMC8546858; doi:10.1128/mBio.02033-21)

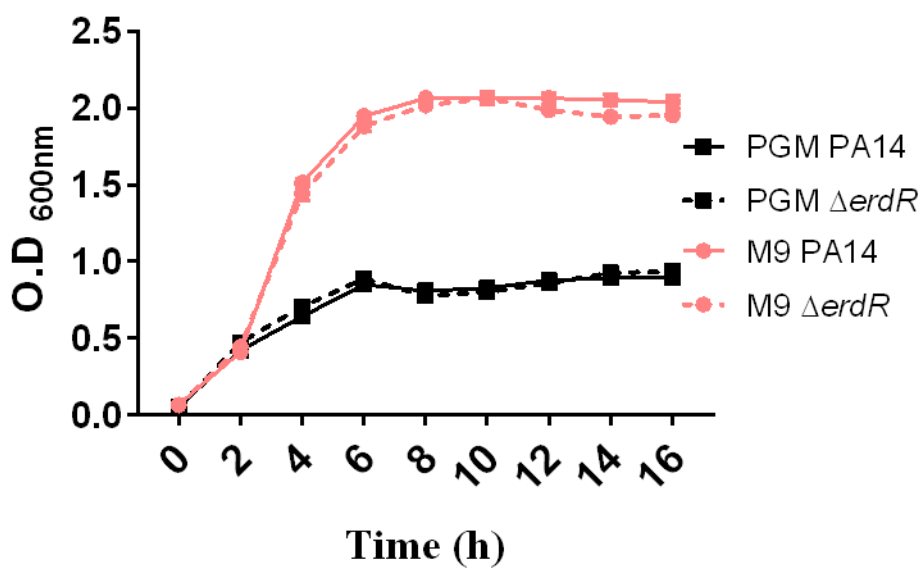

**Figure S6.** Analysis of growth of *P. aeruginosa* PA14 and *erdR* in mPGM broth and M9 broth at 37°C.

Supplement: FIG S6 [file mbio.02033-21-sf006.pdf]
