# Supplementary material for: Foraging Signals Promote Swarming in Starving Pseudomonas aeruginosa
Source: mBio. 2021 Oct 5;12(5):e02033-21. doi: 10.1128/mBio.02033-21 (PMC8546858; doi:10.1128/mBio.02033-21)
Supplement: FIG S7 [file mbio.02033-21-sf007.pdf]

S7

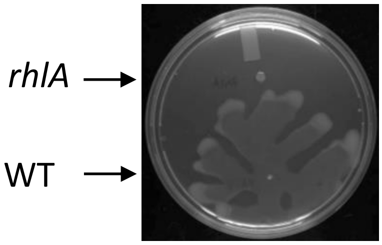


**Figure S7.** Swarming of *rhlA* and WT strains of *P. aeruginosa* on mPGM swarm agar plates with 0.1% ethanol. Imaging was done after 24 hours of incubation at 37°C.
